# Non-drug interventions of traditional Chinese medicine in preventing type 2 diabetes: a review

**DOI:** 10.1186/s13020-023-00854-1

**Published:** 2023-11-14

**Authors:** Jingying Liu, Chun Yao, Yitao Wang, Jinmin Zhao, Hua Luo

**Affiliations:** 1 Macau Centre for Research and Development in Chinese Medicine, State Key Laboratory of Quality Research in Chinese Medicine, Institute of Chinese Medical Sciences, University of Macau, Macao, 999078 People’s Republic of China; 2 College of Pharmacy, Guangxi Medical University, Nanning, 530021 People’s Republic of China; 3 Guangxi University of Chinese Medicine, Nanning, 530001 People’s Republic of China

**Keywords:** Traditional Chinese medicine, Type 2 diabetes, Nonpharmacological intervention, Prevention

## Abstract

Traditional Chinese medicine (TCM) is increasingly used to manage type 2 diabetes and its nonpharmacological interventions are showing potential for preventing type 2 diabetes. This study mainly reviews relevant research. The most mentioned non-drug treatments for preventing type 2 diabetes in TCM are healthy diet, physical activity, emotional therapy, and acupuncture. In most studies, blood glucose status in patients with prediabetes and type 2 diabetes was significantly improved after TCM non-drug interventions, and there was no significant difference between the adverse effect of TCM and control groups or other intervention groups, while the methodological quality of the clinical trials involving TCM generally kept a low level. The effectiveness of TCM in preventing type 2 diabetes has yet to be validated in large randomized controlled trials and the underlying mechanism also needs further exploration.

## Introduction

Diabetes mellitus, more simply called diabetes, manifests as continuous hyperglycemia because any or enough of the insulin cannot be made in the pancreas or efficiently used by the body; it is a severe and common chronic disease [1]. Plasma glucose criteria, either the fasting plasma glucose (FPG) value or the 2-h postload glucose (2hPG) value during a 75-*g* oral glucose tolerance test or glycated hemoglobin (HbA1c) criteria, is the clinical diagnostic indicator of diabetes [2–4] (Table 1).

**Table 1:** Diagnosis of diabetes

Test	Prediabetes		Diabetes
Fasting plasma glucose (mmol/L)	IFG 6.1–6.9 (*5.6–6.9)	IGT < 7.0	≥ 7.0
	or		or
Two-hour plasma glucose (mmol/L)	IFG < 7.8	IGT 7.8–11	≥ 11.1
	or		or
HbA1c (%)	6-6.4 (*5.7–6.4)		≥ 6.5
			or
Random plasma glucose (mmol/L)			≥ 11.1 with symptoms

*IFG* Impaired Fasting Glucose, *IGT* Impaired Glucose Tolerance, *HbA1c* glycated hemoglobin

*Stated in some guidelines, including the American Diabetes Association

Prediabetes is used to describe individuals who exhibit abnormal carbohydrate metabolism but do not yet satisfy the criteria for diabetes and these people present with impaired fasting glucose (IFG) and/or impaired glucose tolerance (IGT) and/or HbA1c 6–6.4% (5.7–6.4%) [5, 6]. With prediabetes, the risk of conversion to diabetes increases, but this can be reduced by active intervention [7–11].

Diabetic patients are more susceptible to developing a series of health problems, which not only influence the quality of life but also threaten the life of patients. State of chronic hyperglycemia can cause serious damage to the body and various organs failure, resulting in disabling and life-threatening health complications and being a major cause of cardiovascular disease, kidney failure, lower limb amputation and blindness. However, appropriate management can help delay or prevent these. Therefore, interventions should be taken to prevent or delay this disease and associated comorbidities [1, 12].

The global prevalence of diabetes appears to be an alarming trend, rising from 10.5% (536.6 million) of people aged 20–79 in 2021 to 12.2% (783.2 million) by 2045 [13]. Besides, it was estimated that about 44.7% (239.7 million) of adults with diabetes did not realize it [14]. Type 2 diabetes, the most common type of diabetes, accounts for almost 90% of the 536.6 million cases [1], making its prevention and treatment imperative. Although contemporary medicines control the diabetic state effectively, it is difficult to reverse the course of the disease. Better, cheaper, simpler treatments with fewer side effects to prevent type 2 diabetes, especially non-drug interventions, deserve to be explored.

Traditional Chinese Medicine (TCM) has been used and developed for more than 2000 years and has become increasingly popular in the East and West over the last several decades [15]. In China, a national survey was conducted to estimate the attitude of patients who receive TCM, Western medicine, and integrative medicine (TCM and Western medicine integrated). The results showed that 71.2% of 2748 participants preferred integrative therapeutic treatments and 18.74% favored the TCM therapeutic method as their favorite [16]. In the US, over 15 million people take herbal remedies or high-dose vitamins. Besides, visits to complementary and alternative medicine far outweighed those to primary physicians, costing more than 34 billion per year [17]. In Australia, in a wide survey targeting older people over 50 years old with chronic diseases, during the past 3 months, 8.8% of the total 2540 participants and 5.1% of 184 individuals with diabetes saw complementary and alternative medicine doctors [18].

In recent years, growing research was conducted on the prevention and management of type 2 diabetes with TCM, and the effectiveness and advantages of TCM in preventing and treating type 2 diabetes were in the process of being confirmed [19]. In addition to herbal remedies, TCM also focuses on diet adjustment and active exercise to prevent type 2 diabetes, which is consistent with modern research. This review summarizes nonpharmacological intervention studies of TCM for preventing type 2 diabetes and compares the similarities and differences between TCM and contemporary medicine, aiming to tap the potential of TCM in this regard and provide new research directions for type 2 diabetes and new non-drug therapy for clinical prevention.

## Type 2 diabetes

Although the causes of type 2 diabetes are not fully understood, several risk factors, including a complex combination of genetic, metabolic and environmental factors, have shown strong relationships with it [20–22], such as family history [23], obesity [24, 25], unhealthy diet [26, 27], physical inactivity [28], and ethnicity [29, 30].

**Fig. 1:**
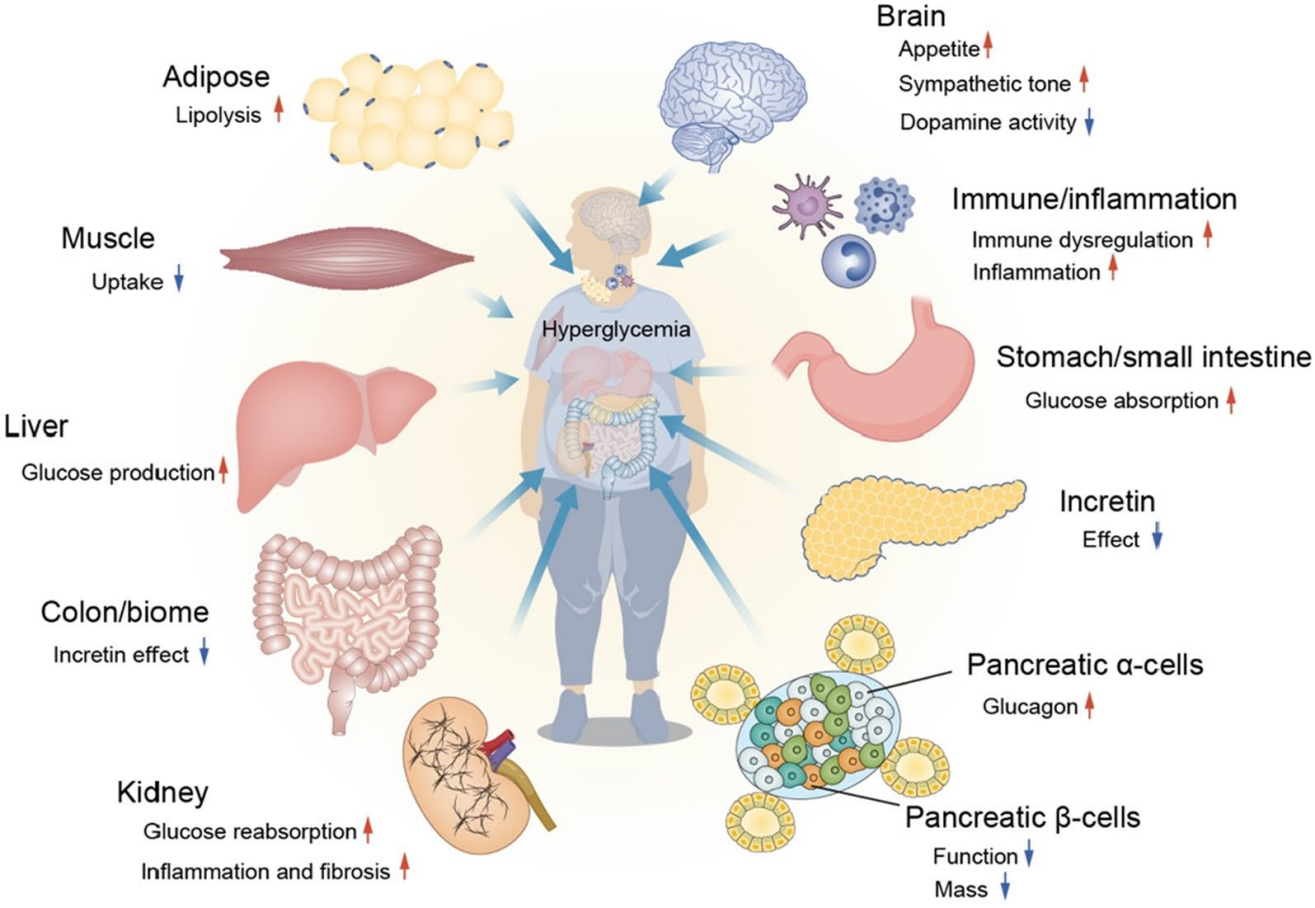
11 defects contributing to hyperglycemia

Insulin resistance and initial hyperglycemia are the primary pathophysiology characteristics of type 2 diabetes, followed by a gradual decline in the ability of pancreatic β cells to produce insulin [31]. In type 2 diabetic patients, adipose tissue, gastrointestinal tract, α-cell, kidney and brain all play important roles in the development of glucose intolerance, besides the traditional triumvirate of insulin resistance in muscle and liver and β-cell failure [32]. These eight core defects were later expanded to 11 mediating pathways that resulted in hyperglycemia [33] (Fig. 1).

Patients with type 2 diabetes present semblable symptoms to those with type 1 diabetes, often characterized by excessive thirst, frequent urination, tiredness, blurred vision, recurrent skin infections, slow wound healing and tingling or numbness in the hands and feet [1, 4]. However, these individuals may live with mild or absent symptoms for several years before diagnosis, making early detection and prevention important.

In ancient China, there was a disease named Xiao Ke, manifesting polydipsia, polyuria and polyphagia, accompanied by emaciation or sweetness of the urine. Due to their similar symptoms, modern TCM doctors regard diabetes as this disease during treating type 2 diabetes [34, 35]. In the last few years, large-scale clinical trials have confirmed that TCM has made progress in hypoglycemic, delaying the transition from prediabetes to diabetes, reducing the risk of diabetic complications and delaying the progression of diabetes complications [19].

## TCM thoughts

Primary prevention focuses on reducing risk factors and preventing the occurrence of type 2 diabetes. Preventing the exacerbation and complication of type 2 diabetes through early detection, early diagnosis and early treatment is the goal of secondary prevention. And delaying the progression of diabetic complications, minimizing morbidity and mortality and maximizing quality of life after a long-term type 2 diabetes or injury are major components of tertiary prevention [34]. In diabetes prevention, primary prevention could gain more preventive benefits than the other two levels of prevention and secondary prevention might obtain greater population benefits than tertiary prevention [36].

TCM holds the similar view of prevention. Huangdi Neijing, as one of four TCM classics, has a famous saying: now, when drugs are employed for therapy only after a disease has become fully developed, when attempts at restoring order are initiated only after a disorder has fully developed, this is as if a well were dug when one is thirsty, and as if weapons were cast when the fight is on. Would this not be too late, too [37]? This reflects one of the most important thinking strongly emphasized by TCM, preventive treatment, including prevention before disease onset and development [38]. For type 2 diabetes, it refers to preventing the onset of prediabetes and type 2 diabetes in healthy individuals, reversing the prediabetic state and preventing it from developing into type 2 diabetes for prediabetic individuals, and maintaining blood glucose levels and preventing further progression, comorbidity and complications for type 2 diabetes patients [39].

## Non-drug TCM interventions

**Fig. 2:**
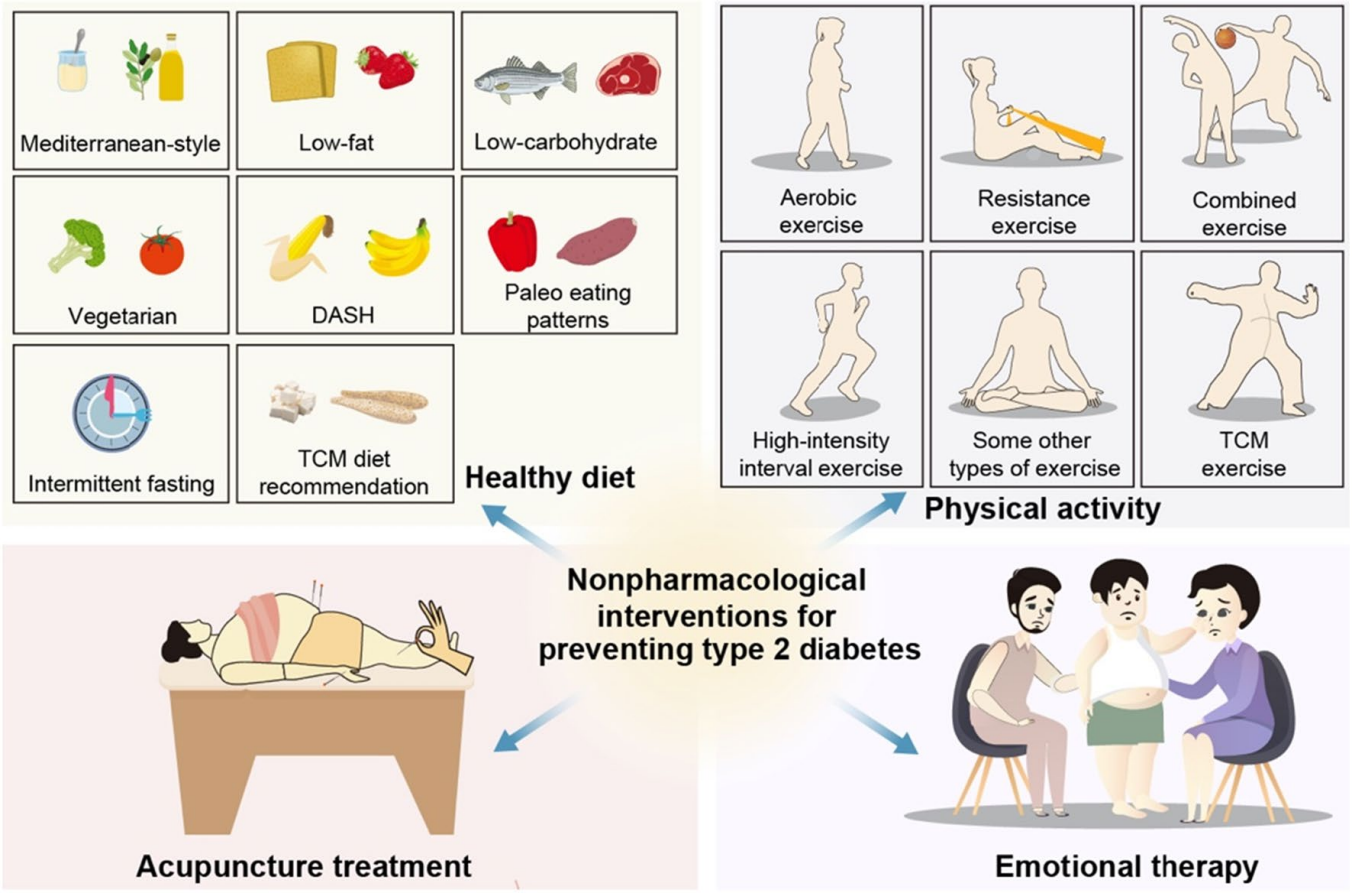
Non-drug interventions for preventing type 2 diabetes

**Fig. 3:**
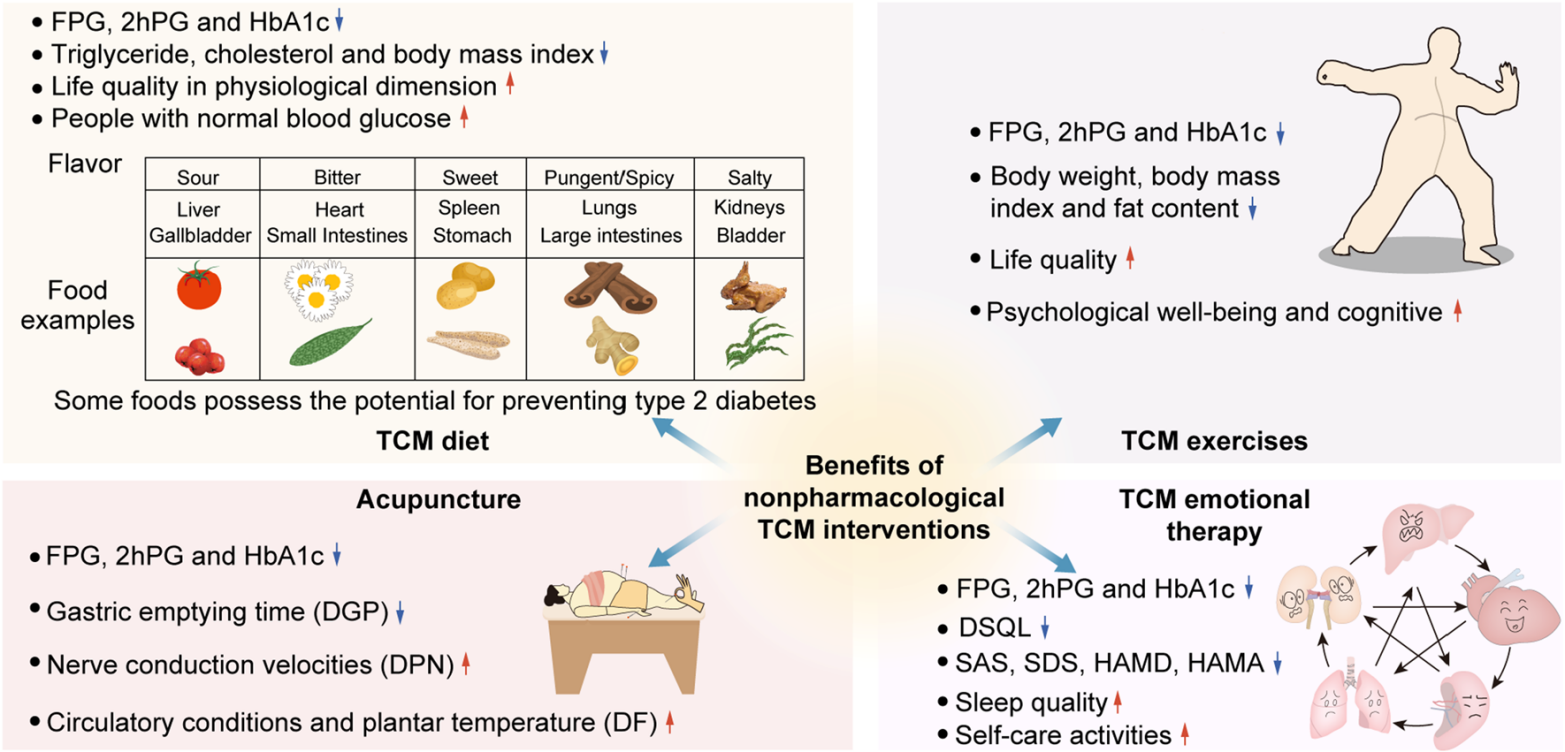
Benefits of non-drug TCM intervention

**Fig. 4:**
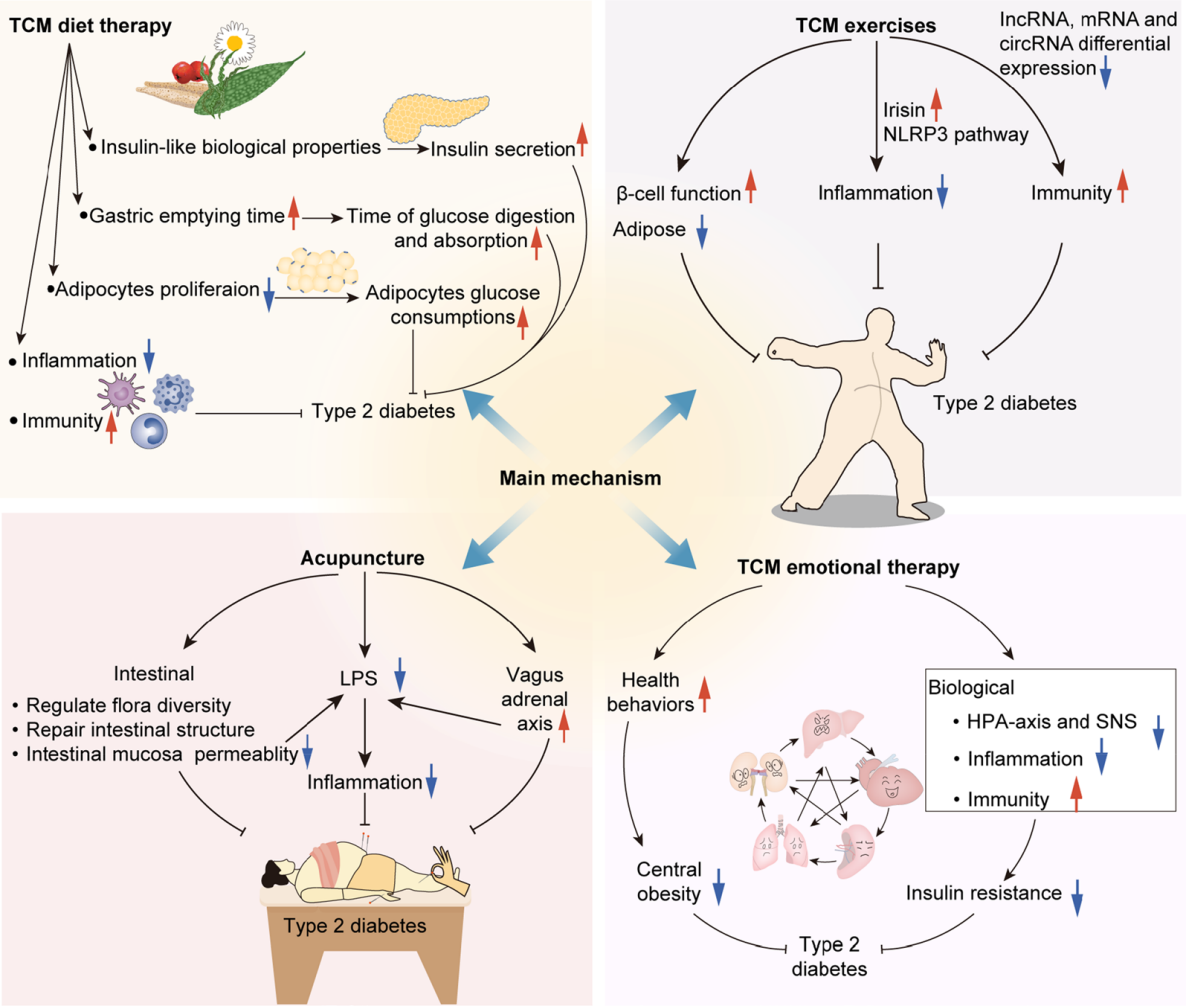
Main possible mechanism of non-drug TCM intervention

Type 2 diabetes is a highly preventable disease through lifestyle modification. Landmark research has shown that prevention can be achieved by physical activity and a healthy diet [40, 41]. The Da Qing Study estimated the effect of a 6-year diet and exercise intervention in people over the age of 45 with IGT. The cumulative incidence of diabetes at 6 years was higher in the control group (67.7%) compared with the three intervention groups (diet only 43.8%, exercise only 41.1%, diet plus exercise 46.0%) [42] and during the 20 years follow-up, 80% of participants in the intervention group and 93% of the participants in the control group had developed diabetes [8]. The Finnish Diabetes Prevention Study, the first randomized controlled trial, aimed to find out whether lifestyle modification can prevent type 2 diabetes alone among people with IGT. The intervention group was given detailed personalized advice involving dietary advice and physical activity guidance. After 4 years, compared with the control group (23%), the cumulative incidence of diabetes was lower in the experimental group (11%) [10], and this result was maintained even if after the lifestyle intervention was ceased [43]. The Diabetes Prevention Program (DPP) compared the efficacy of therapeutic lifestyle changes or metformin and placebo for persons with IGT in the US. After 2.8 years, the incidence of diabetes was 4.8, 7.8 and 11.0 cases per 100 person-years respectively. Both lifestyle interventions and metformin treatment reduced the incidence of diabetes in high-risk groups, but the former was more effective than the latter [44]. This lifestyle or metformin prevention of diabetes can remain for at least a decade [45]. In fact, doing TCM therapy exercises and decreasing the intake of noodles, rice and fruit had been recommended for diabetic patients by TCM doctors, a thousand years before John Rolo, the first person to use diet intervention as a treatment for diabetes [35] (Figs. 2, 3 and 4).

### Healthy diet

Several dietary factors were found to be associated with an increased or decreased risk of type 2 diabetes, regardless of body weight change [40]. Some dietary patterns showed potential for preventing prediabetes or type 2 diabetes. The relevant research available mainly focused on Mediterranean-style, low-fat, low-carbohydrate, vegetarian, dietary approaches to stop hypertension, paleo eating patterns and intermittent fasting. Although diet may vary because of culture, food availability and personal preferences, there are some consensus recommendations on the general principles for type 2 diabetes prevention: a variety of eating patterns are acceptable. Some foods should be encouraged, such as nonstarchy vegetables, unrefined grain products with high natural fiber and nuts, legumes and fish as the source of protein. Some should be limited or reduced, such as sugar, refined grains, red meat, highly processed foods and carbohydrates [46].

In the year 652, Sun Simiao, a famous TCM doctor in the Tang dynasty, suggested that patients with Xiao Ke needed to control drinking and salty food and food made of flour intake. After that, TCM doctors gradually realized that sugar in food worsens the condition and gave some dietary advice to these patients [35]. Based on the theory of nutrition, the theory of TCM and the literature evidence of diet-related chronic disease, some principles and suggestions are put forward for the daily diet of diabetic patients by an expert group in China [47]. It is not difficult to find that the dietary guidelines recommended by TCM are very similar to contemporary medicine, which emphasizes having a bland diet and reducing sugar intake.

However, TCM has its own characteristics, that is, syndrome differentiation and treatment [48], and some food, such as Poria cocos (Fuling) and Dioscorea opposita (Shanyao), also act as Chinese herbal medicine, have great potential in preventing type 2 diabetes [49–51]. From this, daily meals can be combined with TCM health recipes to prevent diabetes. In the Dietary Guidelines for Adults with Diabetes, patients are divided into three main categories. Mori folium (Sangye), Cassiae Semen (Juemingzi) and Nelumbinis Semen (Lianzi) could be adopted for patients with yin deficiency heat excess syndrome to nourish yin and clear heat. Mori Fructus (Sangshen), Lycii Fructus (Gouqizi) and Puerariae lobatae Radix (Gegen) could be used for patients with qi and yin deficiency syndrome to tonify qi and yin. Dioscoreae Rhizoma (Shanyao), Poria (Fuling) and Cinnamomi Cortex (Rougui) are suggested for patients with yin and yang deficiency syndrome [47]. While treating patients with syndrome differentiation, TCM dietotherapy also pays attention to in line with seasonal and local conditions. Besides, TCM also focuses on having regular meals, which refers to the relatively fixed time of three meals a day and additional meals, because regular and quantitative meals can avoid the lethargy of satiety center response caused by excessive hunger, resulting in overeating.

Several clinical trials have proved the ability of TCM dietary therapy to prevent type 2 diabetes. In a randomized controlled trial, 80 patients with IGT were randomly divided into a control group and an intervention group. The former received general nutrient health education, while the latter was guided to take TCM medicated diets according to TCM theory and individual physique. After 1 month and 3 months of treatment, the blood glucose, triglyceride, cholesterol and body mass index in the intervention group were better than those in the control group, and the differences were statistically significant. Furthermore, 37 patients in the intervention group had normal blood glucose after 6 months of treatment, whose outcome was significantly better than that of the control group [52]. Another research explored the effect of the Jianpi Qushi Diet recipe on prediabetic patients with phlegm-dampness constitution. A total of 200 middle-aged patients with prediabetes and phlegm-dampness constitution were selected and randomly divided into two groups. Both groups were given routine health education and hypotensive and lipid-lowering drugs appropriately. Moreover, based on the food exchange method of the control group, the experimental group was given the Jianpi Qushi Diet recipe to replace part of the food. At 6 months and 12 months, the FPG and the 2hPG of the two groups were significantly lower than those before the intervention, and the reduction degree of the experimental group was significantly better than that of the control group, with statistically significant differences [53]. Besides, a meta-analysis of 12 randomized controlled trials including 1178 patients evaluated the influence of TCM diet intervention on blood glucose and life quality of type 2 diabetes. Among them, there were 593 cases in the TCM diet intervention group and 585 cases in the conventional treatment group. The results illustrated that the FPG, 2hPG and HbA1c in the intervention group were significantly lower than that in the control group. Although there was no significant difference between the two groups in psychological and social dimensions, the dietary intervention group was superior to the control group in the physiological dimension of quality of life [54].

TCM dietary therapy shows tremendous potential in improving blood glucose levels in patients with diabetes or prediabetes, the mechanism of action can be considered in the following aspects. First, some TCM medicinal diets may have insulin-like biological properties, which can promote insulin secretion and increase the sensitivity of tissue cells to insulin. Second, some TCM medicinal meals contain rich dietary fiber, so they can delay the gastric emptying time, thereby delaying the digestion and absorption of glucose. Third, the hypoglycemic effect of TCM diet recipes may be achieved by inhibiting the proliferation of adipocytes and promoting the glucose consumption of adipose cells, thus improving insulin resistance. In addition, reducing inflammation in the body and regulating the intracorporeal environment may also be the potential mechanisms [55].

### Physical activity

Physical activity, also an important factor in preventing type 2 diabetes independent of body weight status [56], helps to improve insulin sensitivity [57, 58], reduce visceral fat [58, 59], enhance β-cell function [59] and improve gut microbiota [60]. Based on previous studies, at least 700 kcal/week, equal to a minimum of 150 min of moderate-intensity athletic activities each week like brisk walking, was adopted for DPP [61]. International Diabetes Federation has a similar recommendation for preventing type 2 diabetes by exercising at least 30 to 45 min three to 5 days a week [1]. Multiple types of physical activity have been shown benefit in glycemic management for type 2 diabetes patients, including aerobic exercise, resistance exercise, combined exercise, high-intensity interval exercise and some other types of exercise [62, 63].

In the year 610, Chao Yuanfang, a knowledgeable imperial physician in the Sui Dynasty, proposed that Xiao Ke patients should do Daoyin, a type of TCM therapeutic exercise, and then take 120 steps even up to a thousand steps, and then eat a meal [35]. During the long history of TCM development, a variety of TCM therapeutic exercises have been formed [64], being proven to be suitable for central obesity management, thereby reducing the risk of related diseases [65], and have been recommended for the prevention and treatment of diabetes by Chinese guidelines [66]. Among them, Qigong, Tai Chi, Baduanjin and Yijinjing are most used in clinical experiments and show great potential in the prevention of type 2 diabetes [67–75].

A meta-analysis of nine randomized controlled trials with 485 participants included four types of TCM exercises: Baduanjin, Yijinjing, Tai Chi and Shaolin Kungfu, investigating the effects of these exercises on glycemic control in individuals with prediabetes. The results suggested that among the prediabetes patients, TCM exercises were associated with lower FPG, 2hPG and HbA1c, having potential preventive value for type 2 diabetes [68]. Similar findings were seen in patients with type 2 diabetes. Compared with the comparison group, the TCM exercises group performed better in the glycemic control and observed a significant decrease in FPG and HbA1c [69]. Several studies recommended Tai Chi as a prophylactic and therapeutic exercise prescription for type 2 diabetes [67, 72, 73, 76, 77], which not only improved FPG, 2hPG, HbA1c, triglyceride, high density lipoprotein cholesterol and life quality of people with type 2 diabetes compared with control groups (usual care, regular exercise and clinical conventional therapy) but also better played hypoglycemic effect than that of other aerobic exercises [72, 73, 76, 77]. Moreover, the FPG level was lower in the Qigong group than in that of the resistance exercise group and the HbA1c level of the other aerobic exercise group, and the differences were statistically significant [71]. Baduanjin, another kind of mind-body program, got better improvements in FPG, 2hPG and HbA1c, better gains in body mass index and more positive impact on depression, anxiety and mental health compared with usual care and better improvements in HbA1c, body mass index, depression and anxiety compared with other exercises [75, 78].

A recent randomized controlled trial evaluated the effect of engagement in Baduanjin for prediabetes patients. The results showed that a year of Baduanjin training improved blood glucose, blood lipid profile, body shape and blood pressure and protected against diabetes and atherosclerotic cardiovascular disease of these participants significantly [79]. Besides, Yijinjing combined with elastic band exercise, reducing the body weight, body mass index and fat content and improving muscle function and growth hormone secretion in patients with prediabetes, can delay muscle mass loss and diabetes development [80]. Furthermore, in a randomized clinical trial including 328 patients aged 60 and above with type 2 diabetes and mild cognitive impairment, the Tai Chi Chuan group had higher Montreal cognitive assessment scores compared with the control group and the fitness walking group at 36 weeks, suggesting its potential to prevent cognitive impairment and exacerbation in senile patients with type 2 diabetes [81]. These three recent clinical trials all manifested that adverse events unrelated to the studies did not have statistically significant differences between the TCM exercises group and the other group [79–81].

From a Western perspective, TCM exercises can be classified as a type of light to moderate intensity aerobic exercise that is conducive to metabolism in cells and tissues, cardiac blood reflux, improved glucose utilization and target cell reactivity, reduced glucose resistance, enhanced HbA1c decomposition and accelerated hemoglobin and oxygen binding [71]. Moreover, the potential hypoglycemic mechanism of TCM exercises in prediabetes individuals may be associated with the increase of irisin in blood and inhibition of the NLRP3 inflammatory signal pathway, which helps reduce insulin resistance and inflammation [82]. For type 2 diabetes patients, TCM exercises probably achieved preventive and therapeutic effects by the benefit of immune regulatory function [83]. It may play a role by regulating the abnormal expression of incRNA, mRNA, and circRNA in improving depression symptoms and blood glucose levels of type 2 diabetes patients with depression. [84].

Therefore, TCM exercises provided more variety of choices for type 2 diabetes prevention and could be considered to popularize with their easy to learn and no limit by time and venue nature [71, 79].

### Emotional therapy

An emerging number of the literature suggests that psychological stress and psychiatric disorders may bring on type 2 diabetes [85–87]. The relationship between psychological factors and the occurrence of type 2 diabetes has gotten unprecedented attention and concern from many researchers since English physician Thomas Willis observed that emotional factors such as grief or sadness could cause diabetes [88]. Although the findings were initially inconsistent, the evidence gradually moved to support this link [89].

A series of reviews and meta-analyses have revealed that compared with non-depressed individuals, there was a higher risk of developing type 2 diabetes in depression patients [90–93], and vice versa [94, 95]. In addition, after investigating the relationship between anxiety and the incidence of diabetes, it was found that anxiety may be one of the risk factors for incident diabetes [96, 97]. While prediabetes itself has been linked to the onset of diabetes, a study found that in participants with prediabetes, anxiety could boost its progression to type 2 diabetes [98]. Similar results turned out between prediabetes and depressive symptoms. A recent study illustrated that adults with depressive symptoms accompanied by prediabetes had a higher possibility for type 2 diabetes [99]. Besides, other negative psychological factors, such as posttraumatic stress disorder [100], adverse childhood experiences [101, 102] and job strain [103] are probably correlated to an increased incidence of type 2 diabetes among the initially healthy population.

The mechanisms explaining the link between negative psychological factors and type 2 diabetes can be classified into two main categories. First, psychological stress is associated with adverse health behaviors, involving unhealthy diet, physical inactivity, smoking, sleeping disturbances and medical examination absence, which indirectly increases the risk of type 2 diabetes [86, 90–92, 104]. Second, multiple biological mechanisms are also considered to elucidate this link [86, 92, 95]. Poor health behaviors mentioned before and a side effect of medications can induce weight increase and obesity [105–107]. Furthermore, the hypothalamus-pituitary-adrenal axis and the sympathetic nervous system are activated by chronic stress, increasing the release of cortisol which results in stimulating glucose production, increasing portal and peripheral free fatty acids, decreasing insulin sensitivity and inhibiting insulin secretion [90, 108–111]. Besides, systemic inflammation and immune system also may function in the relationship between negative emotional factors and the increased risk [85, 86, 90]. Although there is not direct evidence of a relationship between negative emotions and the causes of type 2 diabetes, the link between negative emotions and an increased risk of diabetes is increasingly being recognized, suggesting that enhancing mental welling may decrease this hazard and be beneficial to its prevention.

TCM has realized the vital impact of emotions on human health for thousands of years. According to TCM theory, there are seven main categories of emotions: joy, anger, anxiety, pensiveness, sadness, fear and fright. Disorder emotion can cause the dysfunctions of internal organs, developing into different diseases [112], which is gradually supported by some facts. More than 5.9 million people born in Denmark were included in a population-based cohort study that was followed from 2000 to 2016. Cox regression models were used to evaluate the overall hazard ratio and time-dependent hazard ratios for pairs of mental disorders and medical conditions. It was shown that most mental disorders exacerbated the risk of a subsequent illness, with hazard ratios ranging from 0.82 to 3.62, depending on the time the mental disorder was diagnosed [113]. In the etiology of type 2 diabetes, TCM not only emphasizes the important role of lifestyle such as diet and exercise but also recognizes the influence of emotional factors, proposing emotional disorder is one of the causes [114], which is consistent with modern research to some extent [85–87, 90–93, 96–103]. Therefore, emotion regulation is a possible intervention to treat and prevent type 2 diabetes. A new psychotropic treatment model based on rhythm regulation has been applied to the treatment of type 2 diabetes, which not only reduced FPG, 2hPG and HbA1c but also decreased the occurrence of hypothalamic–pituitary–adrenal axis abnormalities, and might forestall the progression of type 2 diabetes and played a preventive role [115]. Although some evidence reported that for type 2 diabetes, the use of antidepressants could be an independent risk factor [107, 116]. Actually, these reports do not conflict with emotional therapy. Because the causality has not been established for some antidepressants at higher doses and longer duration are associated with worsening glucose control. And long-term prospective studies of individual antidepressants rather than class effects should be done.

TCM emotional therapy, without needing to worry about drug reactions, is different from the regulation of emotions by modern drugs. It has special emotional therapy for preventing type 2 diabetes: remind high-risk groups to avoid excessive emotional stimulation, and according to the principle of generation-inhibition in five elements, stimulate existing emotion of the individuals with corresponding emotion [114]. In several clinical trials, patients with type 2 diabetes were randomly split into a control group with routine care and an intervention group given TCM emotional nursing care based on usual care. A number of measures assessing blood glucose levels, self-management ability, quality of survival, negative emotion, symptoms and treatment compliance were compared before and after the trial. These findings suggested that TCM emotional therapy may benefit type 2 diabetics. After the treatment, firstly, the FPG, 2hPG and blood glucose fluctuation in the intervention group were lower than those in the control group, and the differences were statistically significant [117, 118]. Secondly, in comparison with the routine care group, the TCM group had a higher measure scoring on the summary of diabetes self-care activities (SDSCA) and a lower diabetes specific quality of life scale (DSQL) [119, 120]. Thirdly, TCM mental nursing, with lower scores on the self-rating anxiety scale (SAS) and self-rating depression scale (SDS) and a better Pittsburgh sleep quality index (PSQI), demonstrated the ability to improve negative emotions and sleep quality [120–122]. Finally, it was shown that patients in the treatment group had better compliance than those in the usual care group, which was more conducive to maximum therapeutic effect [117, 120, 121]. Besides, there were also some possible advantages for patients with diabetes complications treated by TCM emotional nursing. A few randomized controlled trials have demonstrated that the implementation of emotional nursing of TCM in diabetic patients with anxiety or depression not only contributed to controlling blood glucose, making FPG, 2hPG and HbA1c lower than the control group, but also alleviated the negative feeling. The scores of SAS, SDS, Hamilton anxiety scale (HAMA) and Hamilton depression rating scale (HAMD) were lower than those of the conventional care group [123–127]. For diabetic nephropathy patients, negative emotions were improved and quality of life was promoted after the TCM emotional nursing. And the differences in SAS, SDS as well as DSQL scores between the intervention group and the control group were statistically significant [128, 129]. A study investigated the impact of psychological nursing on diabetic food patients. The results indicated that the HAMA scores, HAMD scores, hospital stays and amputation rates in the intervention group were significantly lower than those in the control group [130]. TCM emotional therapy also manifested a positive significance on the diabetic neurogenic bladder, playing a role in improving clinical symptoms, reducing bladder residual urine volume and relieving anxiety and depression [131]. However, some of the intervention groups in these trials involved TCM massage and exercise, which may have interfered with the outcome, so well-designed studies are needed to illustrate TCM emotional therapy is helpful to type 2 diabetics.

### Acupuncture therapy

The benefits of acupuncture, which has a history of more than 2000 years, are also worthy of attention in type 2 diabetes prevention and treatment [132]. Sun Simiao suggested that moxibustion and acupuncture should be performed in the early stage of Xiao Ke [133]. A classic acupuncture treatment refers to needling 4–12 points and retaining them for 10 to 30 min, and some operations can be conducted to enhance its effect during this time, such as stimulating the needles manually or electrically [134]. For type 2 diabetes treatment, acupuncture therapy was included and recommended in the guidelines for the Prevention and Treatment of Type 2 Diabetes in China (2020 Edition) issued by the Chinese Medical Association for the first time [135].

The primary prevention of type 2 diabetes is mainly targeted at the prediabetic population. A randomized controlled trial evaluated the therapeutic efficacy of point application for senile IGT. Among 64 participants, they were randomly divided into two groups: the control group received dietary intervention while the intervention group was given point application. After 2 courses of treatment, the 2hPG obviously decreased in both groups, with a lower level in the experimental group than in the control group [136]. A pilot randomized study made a comparison between the efficacy of transcutaneous auricular vagus nerve stimulation (taVNS) and pseudo stimulus in patients with IGT. The results showed that there was a significant decline in measures of FPG, 2hPG, and HbAlc in participants treated with taVNS. Only two patients experienced dizziness during or after the treatment and they could completely recover from this uncomfortable feeling after stopping the therapy [137].

However, in healthy volunteers, acupuncture did not significantly reduce random blood glucose levels [138], indicating that acupuncture does not cause hypoglycemia in normal people. Although the available evidence is not strong enough to completely prove that acupuncture intervention in prediabetes reduces the proportion of its turning into type 2 diabetes, with the characteristics of the effectiveness have been proven to some extent, easy to operate and only slight risk of mild side effects, acupuncture is a promising approach to treat prediabetes and prevent type 2 diabetes.

Acupuncture, including typical acupuncture, laser acupuncture, ear acupuncture, herbal acupuncture, electroacupuncture and transcutaneous electrical nerve stimulation, has had the potential for the secondary prevention of type 2 diabetes. It could not only show the hypoglycemic effect, reducing FPG, 2hPG and HbA1c [139–144], but also be helpful for controlling blood glucose, which is better than the control group in continuous glucose monitoring [145]. Besides, auricular acupuncture, improving circulatory conditions and plantar temperature of patients with type 2 diabetes effectively, attributes a preventive effect on the treatment of diabetic foot [146]. It suggested the possibility of acupuncture in preventing the occurrence and exacerbation of diabetic complications.

At present, some clinical evidence for acupuncture improving type 2 diabetes related complications mainly focuses on diabetic peripheral neuropathy [147–151] and diabetic gastroparesis [152, 153]. A recent study of 172 patients with type 2 diabetes induced diabetic peripheral neuropathy evaluated the effects of needle acupuncture, laser acupuncture, or placebo laser acupuncture for them. Comparing needle acupuncture to placebo, sural sensory nerve action potential sural and tibial nerve conduction velocities were significantly improved, and needle acupuncture produced a curative effect earlier than laser acupuncture. In addition, improvement in nerve conduction studies values may prove structural nerve regeneration after acupuncture [148]. For patients with diabetic gastroparesis, both classical needle acupuncture and electroacupuncture have been demonstrated to improve clinical symptoms and reduce gastric emptying time or gastric half-emptying time [152, 153].

Studies have proposed that impaired parasympathetic function existed in prediabetes and was aggravated as the disease developed into diabetes [154]. Immune dysregulation and excessive proinflammatory responses were regulated by the vagus through inflammatory reflex, which is related to the pathogenesis of insulin resistance and the onset of type 2 diabetes [155]. Adequate vagus nerve activity may reduce the incidence of metabolic syndromes, having an important preventative and therapeutic meaning for type 2 diabetes [156]. Thus, acupuncture may prevent type 2 diabetes by driving the vagal-adrenal axis and producing anti-inflammatory effects [157, 158]. Besides, an avalanche of studies turned to understanding type 2 diabetes from gut flora. A research analyzed the fecal bacteria composition of type 2 diabetes and non-diabetic adults by real-time qPCR and tag-encoded amplicon pyrosequencing. Compared with the non-diabetic group, the percentages of phylum Firmicutes and class Clostridia were markedly decreased and class Betaproteobacteria was significantly enriched in the diabetic group. And a significant positive relevance with plasma glucose concentration was shown in the proportions of Bacteroidetes to Firmicutes as well as the proportions of Bacteroides-Prevotella group to C. coccoides-E. rectale group [159]. A plethora of researches have indicated gut microbiota dysbiosis not only predisposed type 2 diabetes but also shared a close relationship with a variety of diabetic complications, suggesting the approaches through modulating gut flora may have preventive and therapeutic effects on type 2 diabetes [160–162]. Some researchers started to interpret the esoteric TCM theory and its mechanism by intestinal flora, and regard gut flora as an approach to revealing TCM core connotation [163]. Acupuncture, as one of the TCM therapy methods, has been found to modulate the composition of intestinal flora in the progression of treating some diseases. [164–166]. Although there were few clinical trials exploring the mechanism of acupuncture in the management of diabetes from the perspective of intestinal flora, animal tests have manifested electroacupuncture was beneficial in increasing the diversity of gut flora and promoting colonic motility, which may be used to explain its hypoglycemic mechanism. [167, 168].

## Conclusion

The preventive ideas and methods of type 2 diabetes and its complications based on TCM theory, showing a strong concordance with contemporary medical views, provide several alternative nonpharmacological interventions, mainly including TCM diet recommendation, TCM exercise, TCM emotional treatment and acupuncture therapy. These TCM non-drug interventions were effective in having hypoglycemic effects in patients with prediabetes or type 2 diabetes, reducing the risk of type 2 diabetes and diabetic complication, delaying its progression and improving the life quality of these patients. There were no significant differences in adverse effects between the TCM and the control or other intervention groups. However, the methodological quality of some studies involving TCM in this article was low and the meta-analysis of TCM mentioned also suggested that most pooled clinical trials were of low methodological quality. Therefore, high-quality, large-scale randomized controlled trials involving safety assessment should be conducted before any definitive conclusions can be come on whether TCM approaches are effective and safe in preventing type 2 diabetes. Besides, given its strong potential to prevent type 2 diabetes, TCM mechanism in this regard deserves further study and exploration.

## Data Availability

Not applicable.

## Abbreviations

DASH: Dietary approaches to stop hypertension
DF: Diabetic foot
DGP: Diabetic gastroparesis
DPN: Diabetic peripheral neuropathy
DPP: Diabetes prevention program
DSQL: Diabetes specific quality of life scale
FPG: Fasting plasma glucose
HAMA: Hamilton anxiety scale
HAMD: Hamilton depression rating scale
HbA1c: Glycated hemoglobin
IFG: Impaired fasting glucose
IGT: Impaired glucose tolerance
LPS: Lipopolysaccharide
SAS: Scores of self-rating anxiety scale
SDS: Self-rating depression scale
taVNS: Transcutaneous auricular vagus nerve stimulation
TCM: Traditional Chinese medicine
2hPG: 2-h postload glucose
